# 3D culture models of Alzheimer’s disease: a road map to a “cure-in-a-dish”

**DOI:** 10.1186/s13024-016-0139-7

**Published:** 2016-12-09

**Authors:** Se Hoon Choi, Young Hye Kim, Luisa Quinti, Rudolph E. Tanzi, Doo Yeon Kim

**Affiliations:** 1 Genetics and Aging Research Unit, MassGeneral Institute for Neurodegenerative Disease, Massachusetts General Hospital, Harvard Medical School, 02129 Charlestown, MA USA; 2 Biomedical Omics Group, Korea Basic Science Institute, Cheongju-si, Chungbuk, 363-883 Republic of Korea

**Keywords:** Alzheimer’s disease, Three-dimensional culture, Amyloid plaques, Neurofibrillary tangles, Induced-pluripotent stem cell, High-throughput drug screening

## Abstract

Alzheimer’s disease (AD) transgenic mice have been used as a standard AD model for basic mechanistic studies and drug discovery. These mouse models showed symbolic AD pathologies including β-amyloid (Aβ) plaques, gliosis and memory deficits but failed to fully recapitulate AD pathogenic cascades including robust phospho tau (p-tau) accumulation, clear neurofibrillary tangles (NFTs) and neurodegeneration, solely driven by familial AD (FAD) mutation(s). Recent advances in human stem cell and three-dimensional (3D) culture technologies made it possible to generate novel 3D neural cell culture models that recapitulate AD pathologies including robust Aβ deposition and Aβ-driven NFT-like tau pathology. These new 3D human cell culture models of AD hold a promise for a novel platform that can be used for mechanism studies in human brain-like environment and high-throughput drug screening (HTS). In this review, we will summarize the current progress in recapitulating AD pathogenic cascades in human neural cell culture models using AD patient-derived induced pluripotent stem cells (iPSCs) or genetically modified human stem cell lines. We will also explain how new 3D culture technologies were applied to accelerate Aβ and p-tau pathologies in human neural cell cultures, as compared the standard two-dimensional (2D) culture conditions. Finally, we will discuss a potential impact of the human 3D human neural cell culture models on the AD drug-development process. These revolutionary 3D culture models of AD will contribute to accelerate the discovery of novel AD drugs.

## Background

Alzheimer’s disease (AD) is the most common neurodegenerative disease worldwide. AD begins with short-term memory impairments, gets worse over time, and culminates in total loss of cognition [1]. Familial, early-onset (<60 years), rare, autosomal-dominant forms of AD (FAD) is caused by fully penetrant mutations either in the amyloid precursor protein (APP), presenilin 1 (PSEN1), or presenilin 2 (PSEN2) genes. Sporadic AD (SAD) is the more common form of the disease, and usually involves late onset owing to multifactorial genetic and environmental risk factors [1–3]. Currently, AD affects 5.3 million people in the United States and the number of AD patients are predicted to increase dramatically over the next decade [4]. However, there is no clear therapeutic option for AD patients yet, except for some symptomatic reliefs [3, 5, 6].

Two key pathological hallmarks of AD are amyloid plaques (a.k.a. senile plaques), and neurofibrillary tangles (NFTs) [4]. The amyloid plaques are extracellular amyloid filaments, composed primarily of small ~4 kDa peptides called β-amyloid (Aβ), which are liberated from the amyloid precursor protein (APP) via sequential proteolytic cleavages by β- and γ-secretase [1, 7, 8]. NFTs are composed of highly phosphorylated forms of the microtubule-associated protein tau (p-tau) [9, 10]. In AD, p-tau dramatically accumulates in the unusual cellular compartments including soma and dendrites, possibly due to an imbalance between the activities of protein kinases and phosphatases [11–13].

For the past decade, AD transgenic mice overexpressing APP or APP/Presenilin (PSEN) with single or multiple familial AD mutations have been used as a standard AD model for basic mechanistic studies and drug discovery [9, 14, 15]. However, these AD transgenic mouse models do not develop clear NFTs nor robust neurodegeneration as observed in human AD patients, despite strong Aβ deposition, synaptic deficits and clear gliosis [9, 14–18]. According to the “amyloid hypothesis,” the accumulation of pathogenic Aβ species, causing amyloid plaques, would trigger a pathogenic cascade that leads to hyperphosphorylation of tau causing NFTs, and ultimately, neuronal death [1, 19–22]. The failures of anti-Aβ therapies in humans, which were highly effective in mouse models, might be explained by the limitation of AD mouse models in comprehensively modeling human AD pathologies [23, 24].

**Fig. 1:**
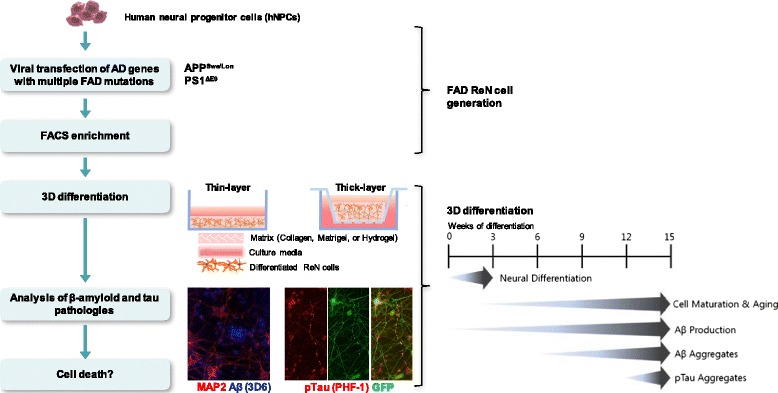
Recapitulation of Aβ and Tau pathology in a 3D human neural cell culture model of AD. Human neural progenitor cells (hNPCs) are virally transfected with APP and/or PSEN1 FAD mutations with either GFP or mCherry as a reporter for viral infection. These cells are enriched based on GFP and/or mCherry signals by FACS, and then differentiated in 3D Matrigel culture systems. Thin-layer (~100–300 μm) culture format is suited for immunostaining analyses and thick-layer (~4 mm) culture format is used for biochemical analyses. In 3D-differentiated hNPCs with FAD mutations, extracellular Aβ aggregates develop ~6 weeks of differentiation and robust increase in tauopathy is evident from ~10–14 weeks of differentiation

Advances in stem cell technology made it possible to generate human neurons with FAD mutations. Induced-pluripotent stem cell (iPSC) technology can even provide human neurons harboring the identical genetic information of AD patients [1, 25–30]. These new exciting human neural cell culture models cast light on making new AD cellular models that can comprehensively recapitulate pathogenic cascades of AD in human brain-like environment. Indeed, we recently showed that the overexpression of APP and PSEN1 with multiple FAD mutations were enough to induce robust Aβ deposition (amyloid plaques), and detergent-resistant, fibrillary p-tau aggregates in human neural cells cultured in our unique Matrigel-based three-dimensional (3D) culture system (Fig. 1), which has not been feasible in AD transgenic mouse models [17, 18, 31, 32]. Our results clearly demonstrate the advantage of human neuronal cells in modeling pathogenic cascades of AD as compared to mouse models.

In this review, we will summarize the current progress in recapitulating AD pathogenic cascades, including Aβ and p-tau pathologies in human neural cell culture models. In addition to new human AD neuronal cell models, derived from fibroblasts, blood cells and CRISPR/CAS9-edited iPSCs, we will focus on how our and other 3D culture technologies were applied to accelerate Aβ and/or p-tau pathologies in human neural cell cultures. Finally, we will discuss a potential impact of these revolutionary human 3D culture models of neurodegenerative diseases on drug-development pipelines.

## Main text

The identification of Aβ as the main component of amyloid plaques resulted in the original formation of the “amyloid hypothesis”, by Drs. Glenner and Wong in 1984 [33], which was later renamed the “amyloid cascade hypothesis” by Drs. Hardy and Higgins [34]. This hypothesis posits that the accumulation of Aβ is the initial pathological trigger in the disease, subsequently leading to the formation of NFTs, neuronal cell death and dementia [2]. However, it has not been easy to fully validate the hypothesis, partly due to the absence of appropriate experimental models. Researchers have taken advantage of FAD-linked APP and/or PSEN1 mutations by developing transgenic mice that express these mutated proteins. Various AD transgenic mouse models have been generated by overexpressing human APP and/or PSEN1 with single or multiple FAD mutations, in which accumulation of Aβ peptides is a common target [9, 14–18]. Since then, AD transgenic mouse models have become the standard model system in vivo for mechanistic studies and AD drug discovery.

It is beyond the scope of this review to discuss all the different AD mouse models, which has already been covered by many reviews including ours [17]. However, although most AD transgenic mouse models recapitulated amyloid plaques and β-amyloid-induced synaptic/memory deficits, none of the AD transgenic mouse models have successfully recapitulated clear Aβ-driven NFT formation nor neuronal death [17]. The limitation of AD mouse models in comprehensively modeling human AD pathologies have led to the failures of anti-Aβ therapies in humans, which were otherwise highly effective in mouse models [23, 24]. 3xTg mice, which express mutant forms of APP, PSEN1 and tau, have shown to develop both plaques and tangle-like pathology [35]. However, this model contains a tau mutation which is associated with frontotemporal dementia (FTD), not AD. It is crucial to note that no mutation has been located in the tau gene in AD and that normal human tau becomes pathological in the disease. Fundamental species-specific differences in genome and proteoforms between mice and humans may preclude the recapitulation of *bona fide* AD pathological events in mouse models. Indeed, adult mice do not express the six human isoforms of tau proteins and endogenous mouse tau seems to interfere with aggregation of human tau proteins [17, 31, 36].

## Recapitulating Aβ pathology in human iPSC-derived neurons

Human neurons derived from AD patients by iPSC technology appear to be an ideal platform for modeling AD in human neuronal environment [25, 26, 29, 30, 37–51]. To date, several research groups have reported the usage of iPSCs in AD modeling, which provide a proof-of-principle for modeling patient-specific AD pathology in human brain-like environment [25–30, 37–42, 48, 49, 52, 53]. These patient-derived AD neurons were mainly generated from FAD patients but also a few from sAD patients. As summarized in our previous review, human iPSC-derived neurons could have successfully recapitulated several pathological features of AD [17]. Most of the FAD neurons carrying FAD mutations in PSEN1 and PSEN2 showed significant increase in the Aβ42/Aβ40 ratio as compared to the non-AD control neurons, confirming that PSEN1 FAD mutations increase pathogenic Aβ42 levels as predicted [1, 25–29]. The iPSC neurons carrying PSEN1 FAD mutations, ΔE9 and L166P were also used as model systems to explore if the PSEN FAD mutations induce “loss of function” of presenilin/γ-secretase in human physiological neurons, which contribute to resolve the controversy over the presenilin/γ-secretase impairment by PSEN FAD mutations [27, 28, 54, 55].

Similar to PSEN FAD neurons, iPSC-derived human neurons harboring APP FAD mutations also showed increases in pathogenic Aβ levels [25, 29, 40, 56]. APP^V717I^ FAD mutation (also termed as London mutation) significantly increased the Aβ42/40 ratio in human forebrain neurons as predicted in non-human model systems [25, 29]. APP E693Δ is a rare autosomal FAD mutation associated with early-onset AD symptoms without Aβ plaques. Consistent with the finding in a different system, AD leads to a reduction in extracellular Aβ levels while inducing the accumulation of intracellular Aβ oligomers in a human iPSC-derived neuronal model [39]. APP duplication (APP^Dp^) is another early-onset FAD mutation, which has been tested in human iPSC-derived neurons [25, 40]. Due to the presence of two copies of the APP gene, these neurons produce high levels of Aβ40 and 42 as compared to other iPSC-derived FAD neurons. Down syndrome (DS) neurons also showed robust increases in total Aβ levels due to the APP gene duplication located on chromosome 21 [57].

In addition to FAD patient-derived neurons, Paquet et al., recently reported the generation of knock-in human neurons harboring heterozygous and homozygous APP or PSEN1 FAD mutations (APP^KM670/671NL^ and PSEN1^M146V^) using modified CRISPR/Cas9 gene editing technology [56]. As predicted, APP^KM670/671NL^ knock-in neurons showed increase in total Aβ levels, while the neurons with PSEN1^M146V^ showed ~2 fold increases in Aβ42/Aβ40 ratio [56]. Since these neurons were originated from non-AD patients, this is a clear demonstration that APP or PSEN1 FAD mutations are sufficient to increase pathogenic Aβ species in human neurons.

Human neurons derived from SAD patients showed variable results in Aβ levels. In general, only a handful of SAD patients showed the increased Aβ levels, which have not been replicable between patients [38–41, 49, 50]. It is not easy to determine whether these variabilities stem from the presence of multiple genetic variants or differential neuronal differentiation conditions due to the lack of isogenic control cell lines. Very limited amount of genetic information is available for these cell lines.

In addition to Aβ accumulation, select AD neurons showed various deficits, which might be triggered by the pathogenic Aβ species. Increased active/total glycogen synthase kinase-3β (GSK3β) levels and enlarged RAB5-positive early endosomes were observed in FAD neurons with APP mutations [29, 40]. APP^E693Δ^ neurons showed elevated endoplasmic reticulum (ER), oxidative stress and altered glycosylation, which can be blocked by Docosahexaenoic acid (DHA) treatments [39]. Abnormal Ca^2+^ influx and the increased susceptibility to cell death have been reported in SAD basal forebrain cholinergic neurons harboring APOε3/ε4alleles [38]. However, it not clear if these deficits are directly connected to the accumulation of pathogenic Aβ. The elevated GSK3β levels in AD neurons were reduced by β-secretase inhibitors, but not by β-secretase inhibitors, which suggest that the accumulation of pathogenic Aβ cannot fully explain the GSK3β changes [40].

## Recapitulating tau pathology in human AD neurons: driven by Aβ or APP-C99?

As previously discussed, AD transgenic mouse model with single or multiple FAD mutations failed to show clear tau/NFT pathologies despite robust Aβ accumulation. As discussed earlier, adult mice do not express tau isoforms as the human brains and even knock in mouse study showed that the presence of endogenous mouse tau inhibit the aggregation of human tau proteins [17, 36].

Patient-derived human neurons can be an excellent alternative model to test if the accumulation of pathogenic Aβ species can induce tau pathology as predicted by the Aβ hypothesis. Indeed, iPSC-derived human neurons with frontotemporal dementia (FTD)-associated tau mutations showed pronounced tau pathology with increased fragmentation of neurites, elevated p-tau immunoreactivity and various cellular deficits including decreased neurite extension, increased cellular stress markers and altered vesicle trafficking [58–60]. Some of these deficits were not observed in parental fibroblast cells, suggesting that FTD tau mutation elicit neuron-specific pathology [59]. These results clearly demonstrate that iPSC-derived human neuronal cultures can be a valid model system for studying tau pathology in vitro.

In the case of AD neurons, not all, but select FAD neurons showed moderate but consistent alteration in p-tau, total tau and/or p-tau/total tau ratio [17]. Israel et al., reported increases tau phosphorylation (p-tau/total tau ratio) in neurons from APP^Dp^ FAD patients and one of the sAD patients, as well as other pathological markers Aβ, p-tau (Thr231) and active glycogen synthase kinase-3β (aGSK-3β), which suggest possible connections among elevated tau phosphorylation, Aβ and GSK-3β activities [40]. However, two Aβ blockers, β-secretase and γ-secretase inhibitors showed contradicting result in reducing tau phosphorylation, which suggests that p-tau increases in these neurons were not solely induced by Aβ species [17, 40]. Muratore et al., also observed the increases in both total tau and p-tau levels in APP^V717I^ FAD neurons [29]. Interestingly, the early treatments with Aβ-specific antibodies reduced the total and p-tau (T231) levels in these neurons, which is a strong evidence that the accumulation of pathogenic Aβ species is responsible for alteration in total and phospho tau levels in APP^V717I^ FAD neurons [29].

Moore et al., also reconfirmed that cortical neurons derived from iPSCs harboring APP^V717I^ or APP^Dp^ duplication mutation showed increases in both total and p-tau (S202/T205, S396 and S404) levels [25]. Interestingly, altered tau metabolism was not observed in neurons carrying PSEN1 FAD mutations (Y111C, M146I and Intron 4) [25]. β-secretase inhibitor or γ-secretase modulator treatments decreased the total and p-tau levels in APP^V717I^, APP^Dp^, DS neurons while the impact of γ-secretase modulator treatments was relatively moderate as compared to β-secretase inhibitor treatments [17, 25]. Interestingly, again, γ-secretase inhibitor treatments did not reduce these tau levels, rather dramatically increased in FAD and even in the control cells [25]. Based on these results, Moore et al., proposed an interesting hypothesis that the total and p-tau change (termed as “tau proteostasis) is regulated by APP metabolism, likely by one of the APP cleavage product, APP-C99, not by Aβ. This hypothesis suggest that BACE1 inhibition therapies can be more effective in reducing p-tau levels in human neurons derived from FAD patients, as compared to other anti-Aβ therapies. The current BACE1 inhibitor clinical trials may be an interesting test for this hypothesis in vivo [61–63]. Moreover, it is not still clear whether the tau proteostasis in these cells is directly relevant to advanced tau/NFT pathology since there is no evidence for tau aggregation nor tau-related pathologies. Also, γ-secretase inhibitor treatments may have also affected other physiological neuronal functions via regulating multiple neuronal substrate cleavages. Further studies will be needed to fully demonstrate the connection among tau proteostasis, APP-C99, and Aβ accumulation [17].

## The limitation of human AD neurons in recapitulating robust AD pathologies

As aforementioned, the human iPSC-derived FAD neurons successfully recapitulated early features of AD including the increases in pathogenic Aβ species. However, these FAD neurons that were not able to fully recapitulate did not show robust extracellular Aβ plaques, Aβ-induced p-tau pathology, NFT pathologies such as aggregated with paired helical filaments (PHFs); neither did they display any signs of neurodegeneration, as predicted in the amyloid hypothesis and observed in AD patients.

Lack of robust AD hallmarks in the iPSC-derived neuronal models might be a result of the following reasons: 1) production of lower levels of pathogenic Aβ species, especially Aβ42, compared to AD patients; 2) insufficient maturation and aging of neuronal cells; 3) fundamental limitation of conventional 2D cell culture systems to mimic complex and dynamic 3D brain environment. The levels of pathogenic Aβ42 in FAD neurons are in the range from 4 to 80 fmol/mg [29, 40, 64]. However, the mean insoluble Aβ42 levels measured in AD brains were ~1,659 pmol/g [65], much higher than the levels observed in iPSC-derived FAD neuronal culture. Thus, the levels of Aβ species generated from FAD iPSC-derived neurons might not be sufficient to form Aβ plaques and other Aβ-triggered pathogenic events. Insufficient neuronal maturation and aging may also contribute to the inability of FAD neurons to reconstitute AD pathologies. As well known, aging is one of the major risk factors of AD and it is still technically challenging to reconstitute mature and possibly aged neuronal culture with human iPSCs. In the case of tau pathology, wild-type human iPSC-derived neurons seem to express low levels of adult 4-repeat (4R) tau isoforms even after 90-days of differentiation [58–60, 66]. 4R tau plays an important role in tau aggregation and NFT pathology in the adult brain, and therefore, low 4R tau levels might also explain why human FAD neurons could not show robust tauopathy with detergent-resistant helical filamentous aggregation [10, 67, 68].

## Accelerating AD pathology using matrigel-based 3D culture system

To overcome the aforementioned limitations of animal models and AD iPSCs-derived neurons, we have recently developed a 3D human neuronal culture model of AD by combining genetically engineered human neuronal progenitor cells (hNPCs) and Matrigel-based 3D culture technology [31, 32]. In order to establish the system, we first generated hNPCs producing high levels of pathogenic Aβ species by overexpressing human APP and PSEN1 with multiple FAD mutations in the ReNcell VM cell line (ReN cells), an immortalized hNPC line that readily differentiates into neurons and glial cells [69]. For FAD mutations, we chose the APP^K670N/M671L^ and APP^V717I^ and PSEN1^ΔE9^ mutation to produce high levels of Aβ species and elevate Aβ42/Aβ40 ratio. Using Fluorescence-activated cell sorting (FACS) enrichment protocols, we generated FAD ReN cell lines which produced ~1000 fold higher levels of Aβ as compared to iPSC-derived FAD neurons (Fig. 1).

In conventional 2D cell culture systems, the secreted Aβ might diffuse into the relatively large volume of cell culture media, and is likely to be removed during regular media changes preventing aggregation of Aβ [17, 31]. To provide the brain tissue-like closed 3D environment which provides a local niche that promotes aggregation of β-amyloid, which can trigger pathogenic cascades, including NFTs, our FAD ReN cells were grown in Matrigel. We chose Matrigel specifically as our 3D matrix because it contains high levels of brain ECM proteins (i.e., laminin, entactin, collagen, and heparin sulfate proteoglycans) and easily solidifies with cells upon moderate thermal change [70]. Cells that were mixed with Matrigel were grown in a thin-layer 3D format (~100–300 μm in thickness) on cell culture vessels with a cover-glass bottom for cellular imaging and in a thick-layer 3D format (up to 4 mm in thickness) using cell culture inserts for molecular and biochemical analyses, respectively (Fig. 1).

Besides the 3D neural cell culture systems being suitable for reconstituting extracellular aggregation of Aβ, they also have advantages in recapitulating in vivo brain environments and can accelerate neuronal differentiation and neural network formation [71–76]. Indeed, we found that our Matrigel-based 3D culture conditions dramatically increased neural differentiation of ReN hNPCs as compared to 2D cultures conditions [17, 32]. More importantly, we found the 3D culture conditions also dramatically elevated 4R adult tau isoforms, which are essential for reconstituting the tau aggregation and NFT pathology [32, 67]. RT-PCR analysis showed 4R/3R tau ratio in 3D-differentiated ReN cells is ~1, which is similar to the ratio in adult human brains [32]. Since we use the same BD Matrigel for 2D and 3D differentiation protocols (only difference is Matrigel concentration), these differences cannot be explained by differential Matrix composition. Together, our results showed that 3D culture conditions have advantages for both accelerating Aβ and NFT pathologies.

The differentiated FAD ReN cells revealed dramatic increases in Aβ40 (~9-fold) and Aβ42 (~17-fold) levels as compared to the control ReN cells, and the Aβ42:Aβ40 ratio was also increased (~5-fold) in ReN cells expressing PSEN ∆E9 mutation [32]. After 6 weeks of 3D-differentiation, not in 2D conditions, we were able to detect clear formation of amyloid plaque pathology: extracellular Aβ deposits were detected by Aβ immunostaining and Amylo-Glo, a Aβ dye and detergent sodium dodecyl sulfate (SDS)-resistant Aβ aggregates were confirmed by biochemical analysis [31, 32].

One of the most interesting aspects of our 3D human neuronal culture model is successful recapitulation of tauopathy without any FTD tau mutation. After ~10 weeks of differentiation, FAD ReN cells showed dramatic increases in phospho tau (pSer199/Ser202/Thr205, pSer396/Ser404) levels in detergent-insoluble fractions from FAD ReN cells without significantly affecting total tau levels. Intracellular accumulation of aggregated, hyperphosphorylated tau proteins was clear in the somatodendritic compartments of FAD neurons, and also we were able to observe the formation of filamentous structures of detergent-insoluble tau proteins [31, 32]. More importantly, inhibition of Aβ generation with β-secretase or γ-secretase inhibitors not only decreased the levels of pathogenic Aβ plaques but also attenuated tau pathology in our 3D cultures, which supports that tau pathology in our system is driven by Aβ accumulation.

Interestingly, we also observed that neurites with high levels of p-tau showed unusual dystrophic morphologies, implying that our 3D models possibly recapitulate dystrophic neurites which is another important pathological feature of AD [32] (*a manuscript under preparation*). The neurite dystrophy, which refer to neuritic sprouting, swollen dendrites and/or axons, has been shown to be the critical neuropathological correlate of dementia in AD [77–83]. Several molecular pathways have been proposed as underlying mechanisms of the neurite dystrophy in AD, based on the studies using AD mouse models and human AD brain samples [78–80, 84, 85]. For example, Hu et al. reported that overexpression of reticulon 3 (RTN3), of which levels were closely related with neurite dystrophy in the brains of AD patients and APP transgenic mice, led to cognitive deficits in mice without Aβ and tau pathologies [85]. In the future studies, it will be interesting to determine potential roles of these proposed pathways on the neurite dystrophy observed in our 3D human neural culture model of AD.

## Accelerating AD pathology using 3D organoid cultures

The self-organizing structures such as cerebral organoids are another way to achieve 3D structures that can facilitate interstitial compartments for Aβ deposition [32]. As previously shown, these brain organoids/neuro-spheroids model can more closely mimic brain structures that are affected in the brains of AD patients [86]. Recently, two groups reported the generation of 3D brain organoid cultures using AD patient-derived iPSCs. Raja et al., used APP^Dp^, PSEN1^M146L^ and PSEN1^A246E^ FAD iPSCs to generate 3D brain organoids/neuro-spheroids that recapitulated AD-like pathologies, including Aβ aggregation, p-tau accumulation at somatodendritic compartments, and endosome abnormalities. The incidence of AD pathology was more robust and consistent as compared to the same cell lines in 2D culture conditions [25, 40, 41]. More importantly, treatment of patient-derived organoids with β- and γ-secretase inhibitors significantly reduces Aβ deposition and tau pathology [32]. Instead of FAD iPSCs, Lee et al. used iPSCs that were derived from 5 different sAD patients’ blood samples [49]. In agreement with Raja et al., they have reported the detection of Aβ and p-tau in brain organoids/neuro-spheroids from sAD iPSCs. They also reported that β- and γ-secretase inhibitors showed less potency in decreasing Aβ levels in neural cells differentiated under 3D culture conditions.

These results clearly demonstrate that 3D organoids culture conditions can accelerate AD pathogenesis in iPSC-derived AD neural cultures, possibly by promoting local Aβ eposition as we proposed in our 3D Matrigel culture models [17, 31, 32]. More importantly, β- and γ-secretase inhibitors significantly reduce p-tau pathology in these organoid cultures, which also support our conclusion that Aβ accumulation is mainly responsible for the robust tauopathy in 3D-cultured AD iPSC-derived neurons. Although it is not shown in these studies, the potential increases in 4R tau isoforms in 3D conditions may also contribute to achieve more robust tau pathology as we showed in our Matrigel-based 3D cultures.

## Application of 3D culture models of AD for drug discovery: looking for a cure in a dish?

**Fig. 2:**
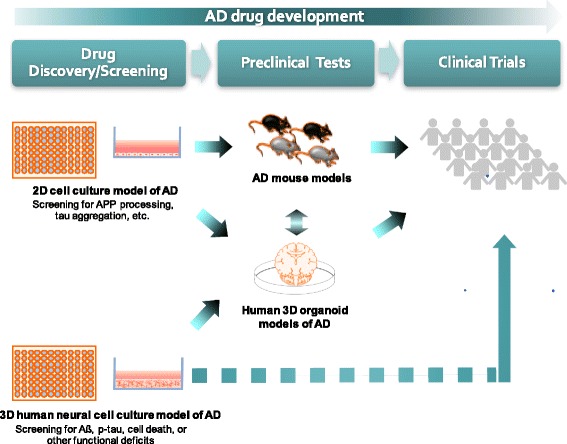
Platform for AD drug screening in a 3D human neural cell culture model of AD. Typically, discovery of new AD drugs goes through three steps: Development and screening drugs in conventional 2D cell culture models of AD (Drug Discovery/Screening); followed by confirming the effects of drugs in AD transgenic mouse models (Preclinical Tests); and after their effects were confirmed both in cell culture and mouse models, drugs are further tested in humans (Clinical Trials). As compared to the conventional 2D cell cultures and animal models, 3D human cell culture models of AD can be more cost-effective and less time-consuming in developing novel AD drugs

In the past decade, AD transgenic mice have been used as a standard preclinical model for testing candidate AD drug targets, which are pre-selected by basic mechanistic studies or by the chemical/peptide library screening using a simple biochemical or cellular model (i.e., APP processing, synthetic Aβ aggregation, tau phosphorylation and etc.) (Fig. 2). The candidate compounds are tested in AD transgenic mice with multiple doses to explore their potential toxicity and the impact on AD pathology, including pathogenic Aβ accumulation, p-tau accumulation and the behavioral and memory deficits. This process takes more than 2–3 years and is relatively expensive. Only small portions of primary candidate compounds can pass through this process. However, a majority of AD drug targets which showed favorable outcome in all the biochemical, cell culture and AD transgenic models, have failed to show efficacy in human clinical trials [23, 87]. A possible explanation for these failures is the limitation of AD transgenic mouse model to fully recapitulate human AD pathology including Aβ-driven NFT and clear neurodegeneration as we discussed earlier.

One of the interesting applications of 3D human neural cell culture models of AD is to use them as a drug-screening platform to accelerate AD drug discovery. 3D culture models of AD can suitably supplement the current drug-development pipeline by providing additional model systems to cross-check the impact of candidate drugs on AD pathogenesis in human brain-like environment (Fig. 2). 3D culture models of AD are also relatively cheaper and faster (6–10 weeks for our 3D culture model; 12 weeks for 3D organoid models) as compared to AD transgenic mouse model. Therefore, they can be easily added to the current drug-development process (Fig. 2). Cross-checking AD drug targets in both human and mouse based models will be helpful to minimize chance of failure in human clinical trials [17, 23]. In addition to the impact on AD pathology, 3D human cellular models can also provide information regarding human-specific toxicity and/or potential side-effects. For some of the candidate drugs that are targeting Aβ driven tau pathology, 3D culture models can be the primary system to assess the efficacy of the drug (Fig. 2).

The most exciting application of the 3D culture models of AD is the unbiased, high-throughput screening (HTS) of new AD drugs in a human brain-like environment (Fig. 2). HTS allows rapid and parallel testing of thousands of compounds in a short time, which is not feasible with AD transgenic mouse models due to their high-maintenance, cost and time-consuming nature. We have previously shown that our Matrigel-based 3D culture model can be easily adapted to HTS formats including 96-well and even in 384-well culture systems [31]. The immortalized and single-clonal ReN cells in our 3D cultures also fit well for large-scale, HTS studies due to their rapid proliferation and stability over multiple passages [17]. Both ELISA and automated immunofluorescence microscopy can be used for measuring AD pathologies in HTS format [31]. Using validated drug libraries like FDA-approved drugs, we can dramatically save time by minimizing the new animal tests since they had already been validated for their toxicity in mouse and human models (Fig. 2). 3D organoids models of AD might be also used to cross-check AD drug targets screened from 3D HTS [53] (Fig. 2).

## Challenges and perspectives

While many advances have been made, challenges still lie ahead to create comprehensive 3D human culture models for AD drug testing and screening. Although our current 3D culture models have successfully recapitulated the AD pathogenic cascades, the overexpression of FAD genes in our 3D culture system, may add additional artificial pathologies, as shown in AD transgenic mouse models [88]. Lack of functional tests such as behavior assessments, is another disadvantage of the current human 3D culture models. Therefore, immediate application of these culture models of AD may be limited to early stage drug development, possibly replacing in vitro cell culture models and conventional cell based HTS assays. The outcome from 3D neuronal HTS assays should provide better predictions of the pharmacokinetic-pharmacodynamic relationship in animal and human trials.

The limited protocols for differentiating forebrain neurons and glial cells might be another technical challenge for reconstituting brain regions in cell culture models, which were mostly affected in AD [17, 31]. Most 2D culture models could not recapitulate complex brain structures and inflammatory components, such as multiple neuronal layer systems, the blood-brain-barrier and microglial components, which would make it hard to assess pharmacodynamic and pharmacokinetic properties that animal models do. In particular, recent studies clearly showed that brain inflammatory components and blood-brain-barrier system play important roles in AD pathogenesis [89–92]. Adoption of advanced cell culture technology, including hydrogel-based 3D culture models, cerebral organoids and microfluidic systems (i.e., organ-on-chip) will be crucial to recapitulating functional brain structures with multiple cell types [17, 32, 76, 86, 93–96].

The 3D organoids models of AD do not rely on the overexpression of FAD genes and have advantages in recapitulating organized brain structures with multiple neural cell types. However, the 3D organoids models of AD might not be suitable for HTS in the current forms due to their well-known heterogeneity [86, 93] and the requirement for longer drug treatments (30–60 days). As we discussed, these 3D organoids models may successfully complement animal preclinical testing (Fig. 2).

The flexible scalability and the use of single-clonal human stem cell lines, which showed rapid and robust AD pathologies, made it easy to fit our Matrigel-based 3D culture model into HTS AD drug screening [17, 31]. However, the use of heterogeneous Matrigel with under-defined and complex protein components, may cause variability for drug testing [70, 97]. Natural hydrogels based on simple extracellular matrix protein components may be potential alternatives for Matrigel [76, 98, 99]. Synthetic hydrogels generally provide much better matrix uniformity and reproducibility than natural hydrogels [94, 100–102]. Further studies will be needed if these synthetic hydrogels can be applicable in 3D human neural cell culture models of AD and the drug testing.

## Conclusions

Developing disease models that fully mirror all, at least key, aspects of the disease is important to understand the disease and ultimately to find cure for it. In AD research, various in vitro 2D cell cultures and animal models, mostly transgenic mouse models, have been widely used. Although they have enormously contributed to AD research, unfortunately, none of these models have successfully reproduced the disease progression found in human AD patients. Recently, we have developed a 3D human neural cell culture model of AD, in which human neural progenitors expressing FAD mutations are grown and differentiated in 3D culture system, which mimics the brain environment [31, 32]. In our 3D culture system, but not in 2D, these neurons successfully generated extracellular aggregation of Aβ and tauopathy driven by the Aβ accumulation, which was not feasible in AD transgenic mouse models. Recently studies also showed that 3D organoids cultures were successfully adapted to accelerate AD pathogenesis in iPSC-derived AD neural cultures. These exciting 3D human cell culture models of AD will serve to facilitate the development of more precise human cellular models of AD for basic mechanistic studies and contribute to the discovery of novel AD drugs.

## Abbreviations

2D: Two-dimensional
3D: Three-dimensional
AD: Alzheimer’s disease
APP: Amyloid precursor protein
Aβ: β-Amyloid
FAD: Familial Alzheimer’s disease
hNPCs: Human neural progenitor cells
HTS: High-throughput screening
NFT: Neurofibrillary tangle
PSEN: Presenilin
ReN: ReNcell VM
SAD: Sporadic Alzheimer’s disease
